# Equine pericardial roll graft replacement of infected pseudoaneurysm of the aortic arch

**DOI:** 10.1186/1749-8090-7-45

**Published:** 2012-05-14

**Authors:** Hiroshi Kubota, Hidehito Endo, Mio Noma, Hiroshi Tsuchiya, Akihiro Yoshimoto, Mitsuru Matsukura, Yu Takahashi, Yusuke Inaba, Kenichi Sudo

**Affiliations:** 1 Department of Cardiovascular Surgery, Kyorin University, 6-20-2, Shinkawa, Mitaka, Tokyo, 181-8611, Japan

**Keywords:** Equine pericardium, Biomaterial, Infection, Aortic aneurysm, Surgery

## Abstract

Resection of the infected aorta, debridement of the surrounding tissue, in situ graft replacement, and omentopexy is the standard procedure for treating infected aortic aneurysms, but the question of which graft material is optimal is still a matter of controversy. We recently treated a patient with an infected thoracic aortic aneurysm. The aneurysm was located in the proximal aortic arch. Because the patients had previously undergone abdominal surgery, the aortic arch were replaced in situ with a branched equine pericardial roll grafts. The patient is alive and well 23 months after the operation.

## Background

**Figure 1:**
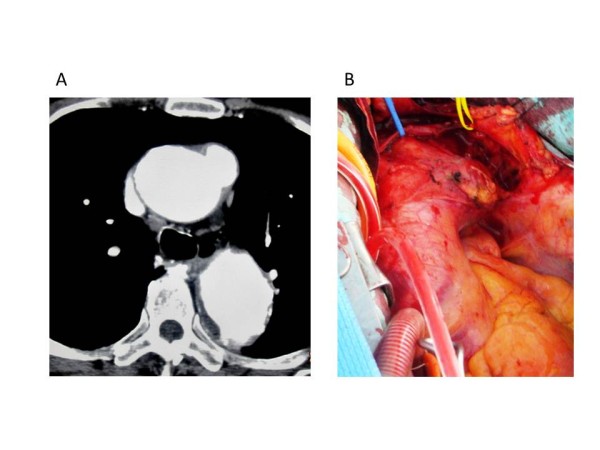
**A, B. Preoperative computed tomography scan and intraoperative photograph.** The aneurysm was located in the proximal portion of the aortic arch. It was visible opposite the orifice of the brachiocephalic artery.

A 79-year-old male came to our hospital with a chief complaint of fever of unknown origin and low back pain. His leukocyte count was 9300/μl, and his serum C-reactive protein level was elevated to 19.8 mg/dl. After three weeks of intravenous antibiotic therapy in another department with Cefotiam 3 g/day, the laboratory data indicating inflammation had become normal. Because computed tomography revealed an abdominal aortic aneurysm measuring 69 mm in diameter and a small pseudoaneurysm in the proximal aortic arch, the patient was referred to our department. In view of the risk of rupture of the abdominal aortic aneurysm, Y-graft replacement was performed first. The abdominal aneurysm was atherosclerotic, but there was no evidence of infection. Postoperative computed tomography showed rapid enlargement of the pseudoaneurysm of the proximal aortic arch (Figure 1-A). He was diagnosed as an infected thoracic aortic aneurysm. The aortic arch replacement was performed.

## Methods

### Preparing a three branched equine pericardial sheet

**Figure 2:**
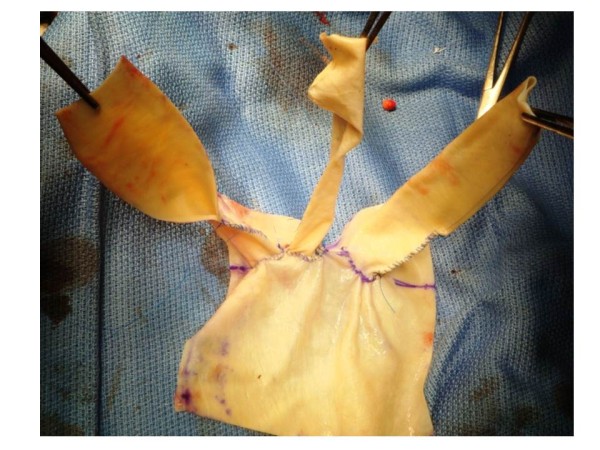
**Three-branched pericardial sheet.** Three holes, having a diameter of 10 mm, 10 mm, and 15 mm, respectively, were made. The holes were made 5 mm apart, and the last hole was made 20 mm from the edge of the sheet. Three rectangular sheets were cut from another pericardial sheet, and each of them was sutured to the circumference of a hole in the pericardial sheet and formed into a cylinder by continuous suturing with 5–0 polypropylene.

A branched equine pericardial sheet was made on another sterile operative table simultaneously to the operation. A 10 cm × 10 cm equine pericardial sheet (XGA-400; Edwards Lifesciences, Irvine, CA, USA) was prepared. Three holes in a row were made. A 10-mm hole each was created for the left subclavian artery and left common carotid artery, and a 15-mm hole for the brachiocephalic artery. The holes were made 5 mm apart, and the last hole was made 20 mm from the edge of the sheet. Three rectangular pericardial sheets were cut from another pericardial sheet. Each of them was sutured to the circumference of a hole and formed into a cylinder by continuous suturing with 5–0 polypropylene. To determine the appropriate length of the graft to avoid the kinking of the branches, these sutures to roll up the branches were stopped 20 mm from origin of the branch and the remnants were left open (Figure 2).

### Operative procedure

**Figure 3:**
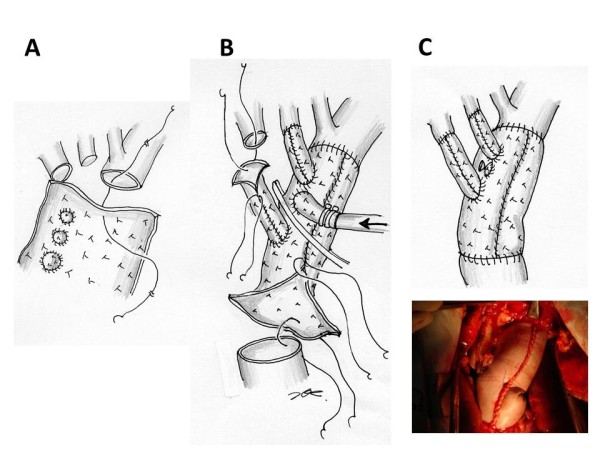
**Operative procedure.A.** The aorta was transected between the left subclavian artery and the left common carotid artery. A branched equine pericardial sheet was anastomosed to the transected aorta. **B.** The sides of the pericardial sheet were sutured continuously to form a cylinder. The third distal branch was cut to obtain adequate length and anastomosed to the left common carotid artery. The second middle branch was used as an inflow root of the cardiopulmonary bypass. The main roll graft was clamped, and antegrade perfusion was restored. **C.** The first proximal branch was anastomosed to the brachiocephalic artery. Finally, the proximal anastomosis was performed.

The operation was performed on February 13, 2010. The pericardium was opened through a median sternotomy and revealed a moderate amount of muddy reddish brown pericardial effusion with pus laver. When the patient’s tympanic membrane temperature had decreased to 20 degrees centigrade, the aortic arch was opened under circulatory arrest. A saccular pseudoaneurysm was identified in the proximal aortic arch, opposite the orifice of the brachiocephalic artery, and a reddish brown thrombus was found inside the aneurysm (Figure 1-B). The aorta was transected between the left subclavian artery and the left common carotid artery. A side of the branched equine pericardial sheet was anastomosed with a 4–0 polypropylene continuous suture (Figure 3-A). When the corners of the pericardium met, the same thread was used continuously to form the sheet cylindrically. Because the diameter of the transected aorta was 30 mm, 10 cm x 10 cm pericardial sheets were used as is. The third distal branch was cut to obtain adequate length and anastomosed to the left common carotid artery. The second middle branch was not used to reconstruct a vessel because native subclavian artery could be preserved, but it was used as an inflow root of the cardiopulmonary bypass. The main roll graft was clamped, antegrade perfusion was restored from the middle branch (Figure 3-B). While warming, the first proximal branch was anastomosed to the brachiocephalic artery. After completing the proximal anastomosis, the aorta was de-clamped (Figure 3-C). The aortic cross clamp time was 120 min, and the circulatory arrest time was 60 min. The intermittent pressure-augmented retrograde cerebral perfusion was used as additional brain protection during deep hypothermic circulatory arrest, and biological glue containing vancomycin was sprayed on the grafts and anastomoses [1].

Written informed consent was obtained from the patient for publication of this report and any accompanying images.

## Results

Intraoperative microscopic examination of the thrombus in the pseudoaneurysm revealed a large number of leukocytes. The pericardial effusion also contained a large number of leukocytes.

**Figure 4:**
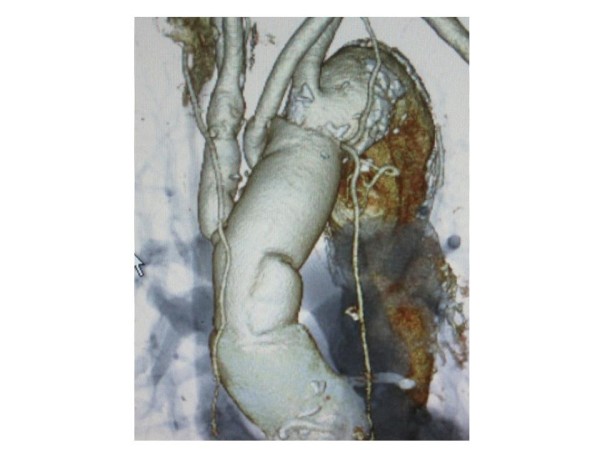
**Postoperative 3-dimension computed tomography.** There is no stenosis or dilatation of the branches.

Culture results from blood and intraoperative specimen were negative. The patients recovered from surgery well and had no neurological deficits. Treatment with an antiplatelet drug was started. Considering the effectiveness of the initial treatment with wide spectrum antibiotics, we targeted as the second series of the antibiotic therapy, not to the MRSA but common microorganism. Dibekacin sulfate (50 mg/day) and cefazolin sodium (4 g/day) were administered intravenously for one week. Continuously, Biapenem (600 mg/day) and clindamycin (600 mg/day) were administered for three weeks and terminated because of systemic drug eruption. The patient’s leukocyte count and C- reactive protein level gradually decreased to within the normal range. As of 24 months after surgery, the patient is well without recurrence of any evidence of the infection. Postoperative computed tomography demonstrated well functioning roll grafts without stenosis, dilatation, or thrombus formation (Figure 4).

## Discussion

Dacron grafts, rifampicin-soaked Dacron grafts, and homografts are the choices to cure infected aortic aneurysm [2-4]. Although cryopreserved homografts are excellent material to treat infected aortas, it is difficult to be in time for urgent operation. Yamamoto et al. described successful in situ replacement of the thoracic descending aorta with an equine pericardial roll graft for an aortobronchial fistula caused by the infection due to α–streptococcus [5]. Omentopexy was not performed in their patient, because omental mobilization was considered impossible due to a past history of laparotomy for an esophageal hiatal hernia. In our case, we also did not use the omentum because abdominal adhesions were expected and the lesion was thought to be too distant to wrap the graft. Yamamoto et al. also described two cases of successful in situ replacement with an equine pericardial roll graft to treat a ruptured infected abdominal aortic aneurysms [6]. In presented case, no pathogen was detected. It may have been due to the long preoperative period of intravenous antibiotic therapy. The type of pathogen also affects the prognosis. A case who showed the colonized and damaged inner layer of the equine pericardial roll graft by methicillin-resistant *Staphylococcus aureus* was reported [7].

Czerny et al. reported excellent result of the bovine pericardial tube graft to treat prosthetic graft or endovascular graft infection in 15 patients. They concluded that treatment of graft infections after operation or endovascular treatment of thoracic, thoracoabdominal, and abdominal aortic diseases by complete removal of the infected prosthetic material and extensive debridement as well as orthotopic vascular reconstruction using self-made xenopericardial tube grafts as neoaortic segments provides excellent results with regard to durability and freedom from reinfection and reoperation [8]. They also mention that this new concept is an additional alternative to cryopreserved homografts that extends the armamentarium for treating patients with highly complex conditions. Considering these excellent result of self-made xenopericardial tube graft as “rescue” procedure, we propose a question that why not use the xenopericardial tube graft for “initial” treatment.

However, As far as we investigated, we could not find any reports of aortic arch reconstruction with a self-made branched equine pericardial roll graft. A pericardial sheet is soft but firm and easy to suture. It was made to be cylindrical not preoperatively but intraoperatively to obtain a good operative field. The diameter of the roll graft was easy to match to the transected aorta. Because the side length of the pericardium was 10 cm, an each margin to sew up was calculated as 10–3 π/2 = 0.3 cm. The graft dilatation, stenosis, mural thrombus formation, and recurrence of the infection are concerns during long-term follow up. Enhanced computed tomography may be the most suitable examination for follow-up. The need for treatment with antiplatelet agents is a matter of controversy. We propose that, patients who have undergone surgical reconstruction of the arch vessels, be treated with an anticoagulant or antiplatelet drug to prevent strokes and graft stenosis due to mural thrombi.

## Conclusion

Because this technique is simple and less invasive than the standard procedure, it may have the potential to serve as one of the choices of treatment for infected aneurysms of the thoracic aorta. By accumulating clinical cases, when its long-term durability will be confirmed, it may demonstrate the advantages of xenopericardial branched graft as one of the choices of treatment for infected aortic aneurysms.

## Competing interest

The authors declare that they have no competing interests.

## Authors’ contributions

HK, HE, MN, HT conceives of the study, and participated in its design and coordination. AY, MM, YT, YI and SK participated in the sequence alignment. All authors read and approved the final manuscript.
